# Gene Transfer in *Leptolyngbya* sp. Strain BL0902, a Cyanobacterium Suitable for Production of Biomass and Bioproducts

**DOI:** 10.1371/journal.pone.0030901

**Published:** 2012-01-24

**Authors:** Arnaud Taton, Ewa Lis, Dawn M. Adin, Guogang Dong, Scott Cookson, Steve A. Kay, Susan S. Golden, James W. Golden

**Affiliations:** Division of Biological Sciences, University of California San Diego, La Jolla, California, United States of America; Kansas State University, United States of America

## Abstract

Current cyanobacterial model organisms were not selected for their growth traits or potential for the production of renewable biomass, biofuels, or other products. The cyanobacterium strain BL0902 emerged from a search for strains with superior growth traits. Morphology and 16S rRNA sequence placed strain BL0902 in the genus *Leptolyngbya*. *Leptolyngbya* sp. strain BL0902 (hereafter *Leptolyngbya* BL0902) showed robust growth at temperatures from 22°C to 40°C and tolerated up to 0.5 M NaCl, 32 mM urea, high pH, and high solar irradiance. Its growth rate under outdoor conditions rivaled *Arthrospira* (“*pirulina”* strains. *Leptolyngbya* BL0902 accumulated higher lipid content and a higher proportion of monounsaturated fatty acids than *Arthrospira* strains. In addition to these desirable qualities, *Leptolyngbya* BL0902 is amenable to genetic engineering that is reliable, efficient, and stable. We demonstrated conjugal transfer from *Escherichia coli* of a plasmid based on RSF1010 and expression of spectinomycin/streptomycin resistance and *yemGFP* reporter transgenes. Conjugation efficiency was investigated in biparental and triparental matings with and without a “elper”plasmid that carries DNA methyltransferase genes, and with two different conjugal plasmids. We also showed that *Leptolyngbya* BL0902 is amenable to transposon mutagenesis with a Tn*5* derivative. To facilitate genetic manipulation of *Leptolyngbya* BL0902, a conjugal plasmid vector was engineered to carry a *trc* promoter upstream of a Gateway recombination cassette. These growth properties and genetic tools position *Leptolyngbya* BL0902 as a model cyanobacterial production strain.

## Introduction

Great interest is being focused on photosynthetic microorganisms for their ability to convert solar energy and CO_2_ into fuels and other bioproducts. Cyanobacteria provide an excellent platform for the production of renewable biofuels and other products [1], [2]. Cyanobacterial carbohydrate and lipid metabolism has been studied by several laboratories but much remains to be understood [3]–[5]. Cyanobacteria typically accumulate glycogen and polyhydroxyalkanoates rather than lipids as stored energy, but their photosynthetic membranes are rich with glycolipids and they naturally produce hydrocarbons [4], [6], the major constituents of gasoline, diesel, and jet fuel [7].

Cyanobacteria have been a major component of our biosphere for over 2.5 billion years [8]. Architects of our atmosphere, these photosynthetic organisms still play an essential role in biogeochemical transformations, particularly in the oceans where they may account for more than 50% of phytoplankton biomass and primary production [9]. With a wide range of metabolic capabilities and few nutritional demands, cyanobacteria live in diverse environmental conditions [8]. Some fix nitrogen, reducing the need for nitrogen fertilizer and the associated production of nitrous oxide, a major greenhouse gas [10], [11]. Most species tolerate high pH and some tolerate high salt concentration, conditions that help to control contaminants and predators in outdoor ponds. Certain cyanobacteria produce a mucilaginous envelope for protection against predators and desiccation. Regulation of photosynthetic antenna complexes called phycobilisomes allow cyanobacteria to adapt to changes in light quality and to extremely low light levels [12], and extracellular and intracellular screening pigments protect them against high light or UV radiation [13]. Cyanobacteria usually have higher growth rates than other phytoplankton under low light [14]. In addition to their physiological and ecological variety, cyanobacteria are also diverse in terms of morphology, including multicellular filamentous species that may bioflocculate or float to the surface of a pond for easier harvesting. These characteristics reflect their genetic diversity and make them good sources for gene mining.

As prokaryotic, gram-negative bacteria, cyanobacteria are easy to manipulate genetically. Extensive genetic tools have been developed for a variety of different species. DNA can be introduced into cyanobacteria by transformation, conjugation, and electroporation and then propagated in the strain if carried on a replicating plasmid or if integrated into the host chromosome [15], [16]. However, genetic approaches have been developed for only a limited number of model strains used to investigate fundamental processes such as photosynthesis, nitrogen fixation, and circadian rhythmicity [16]–[18]. Productivity, particularly outside of the highly regulated environment of the laboratory, and the ability to grow in a wide range of ecological conditions were not determining factors in the selection of these strains for laboratory studies.

Research featuring genetically engineered cyanobacteria for the production of liquid biofuels including ethanol [19], isobutyraldehyde and isobutanol [20], and free fatty acids [21] has recently flourished. Although using cyanobacteria as cell factories has become more common, studies are still carried out with standard laboratory model organisms rather than with potential production strains. For their desirable growth qualities, much consideration has been given to strains of the genus *Arthrospira* (“*pirulina*”, which are grown at industrial scale mostly as a nutritional supplement. However, several attempts to transform *Arthrospira* strains have had only limited success [22], [23], and to our knowledge there is no reliable genetic system for the stable transformation of *Arthrospira* spp.

We have identified and characterized the cyanobacterial strain *Leptolyngbya* sp. strain BL0902 (hereafter *Leptolyngbya* BL0902), which emerged from a screen of cyanobacterial strains for superior growth traits, and show that it is amenable to genetic manipulation. *Leptolyngbya* BL0902 has good growth characteristics when compared to two common outdoor production strains of the genus *Arthrospira*. We show that *Leptolyngbya* BL0902 can receive and maintain conjugal shuttle vectors, express an antibiotic resistance gene and a *yemGFP* reporter gene, and be subjected to transposon-tagging mutagenesis.

## Results

### Morphological description and identification

**Figure 1:**
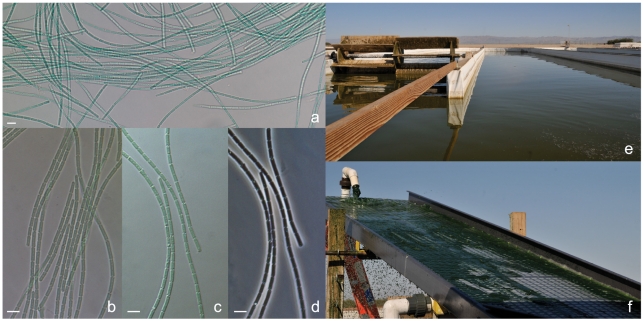
Photomicrographs of wild-type *Leptolyngbya* BL0902 (a, b, c, and d), and its growth in a microalgae farm in Imperial Valley, California (e and f). Bright field (a and b), differential interference contrast (c), and phase contrast microscopy (d). 1-acre paddle-wheel raceway microalgae pond (e) and filamentous cyanobacteria collected with a vibrating screen (f). Scale bars, 5 µm.

*Leptolyngbya* BL0902 is a filamentous cyanobacterium without heterocysts, akinetes, or true or false branching; filaments are composed of single trichomes (chains of cells) that are straight to wavy and lack conspicuous motility. Trichomes are cylindrical and usually unsheathed, but a very thin hyaline sheath might be observed at trichome breakage; necridic cells are absent. Trichomes are slightly constricted at the cross-walls; cells are 1.3 to 3.3 times longer than wide with an average size of 1.42±0.15 (1.12 – 1.66) µm wide, 3.11±0.57 (2.09 – 4.18) µm long; and end cells are rounded. The cytoplasm is homogeneous with a few granules but no gas vesicles (Fig. 1).

### Molecular identification based on 16S rRNA gene and ITS

Based on 16S rRNA data, *Leptolyngbya* BL0902 may be considered novel. The top hit identified by BLAST was *Spirulina laxissima* SAG 256.80 with 97.8% identity (Table 1) and to which no detailed morphological description is associated, leaving the possibility of misidentification. The uniqueness of *Leptolyngbya* BL0902 was verified by the Internal Transcribed Spacer (ITS) between the 16S and 23S rRNA genes, which shared only 89% identity with the first hit identified by BLAST (Table 2).

**Table 1:** BLAST results obtained by querying the 16S rRNA gene of *Leptolyngbya* BL0902 with GenBank, and geographical and ecological origins of the hits.

Description^a^	Accession	Query coverage	Score	E-value	% identity	Origin of the strain or clone	Reference
*Spirulina laxissima* strain SAG 256.80	DQ393278	100%	2257	0	97.8	Lake Nakuru, natron lake, Kenya	-
Uncultured bacterium clone DP10.3.11	FJ612370	100%	2239	0	97.3	Dongping Lake, China	-
Uncultured bacterium clone GBI-83	GQ441263	100%	2167	0	96.4	Marine microbial mats from a sandy intertidal beach, Schiermonnikoog, The Netherlands (53.48 N 6.13 E”	-
*Leptolyngbya* sp. strain 0BB30S02	AJ639892	100%	2161	0	96.5	Bubano Basin, Imola, Italy	[60]
Pseudanabaenaceae cyanobacterium DPG1-KK5	EF654067	100%	2161	0	96.4		[61]
*Phormidium* sp. strain 195-A12	EU282429	100%	2159	0	96.7	Siberian permafrost, borehole 1/95, 2.4–2.45 m, Kolyma Lowland, Siberia, Russia	-
Uncultured bacterium clone GBII-87	GQ441350	100%	2158	0	96.6	Marine microbial mats from a sandy intertidal beach, Schiermonnikoog, The Netherlands (53.48 N 6.13 E”	-
*Leptolyngbya antarctica* ANT.ACE.1	AY493588	100%	2152	0	96.4	Ace lake, Vestfolds hills, Antarctica	[62]
Uncultured cyanobacterium clone R8-R56	DQ181691	100%	2146	0	96.3	Lake Rauer 8, Rauer Island, Antarctica	[63]
*Phormidium* sp. strain MBIC10025	AB183566	95%	2145	0	97.3	Pacific Ocean, 27.03N-141.54E	-
*Leptolyngbya* sp. strain 0BB19S12	AJ639895	100%	2145	0	96.3	Bubano Basin, Imola, Italy	[60]
*Leptolyngbya* sp. strain MX1	GQ848193	100%	2134	0	96.1	Lake Taihu, Shangai, China	-
*Leptolyngbya* sp. strain PCC 7104	AB039012	100%	2128	0	96.1	Rock at shoreline, Montauk Point, Long Island, New York, U.S.A	[24]
*Leptolyngbya* sp. strain 0BB24S04	AJ639893	100%	2128	0	96	Bubano Basin, Imola, Italy	[60]
*Phormidium* sp. strain SAG 61.90	EU624415	100%	2128	0	95.9	River Meuse near Tihange, Belgium	[61]
Uncultured cyanobacterium clone A132	DQ181668	100%	2124	0	96	Ace lake, Vestfolds hills, Antarctica	[63]
*Leptolyngbya nodulosa* UTEX 2910	EF122600	100%	2117	0	95.7	South China Sea	[64]
Uncultured *Leptolyngbya* sp. clone EHFS1_S05b	EU071483	100%	2117	0	95.7	ESTEC HYDRA facility	-
Uncultured cyanobacterium clone AS-45-2	FJ866611	100%	2117	0	95.7	Submerged sinkhole	-
*Leptolyngbya* sp. strain 0BB32S02	AJ639894	100%	2111	0	95.7	Bubano Basin, Imola, Italy	[60]
*Oscillatoria neglecta* IAM M-82	AB003168	100%	2100	0	95.9		[65]
*Oscillatoria* sp.	AJ133106	100%	2091	0	95.4	Lake Loosdrecht, The Netherlands	-
*Leptolyngbya* sp. Kovacik 1999/1	GQ495618	100%	2074	0	95.9	Biofilm from interior wall, Cartusian Monastery Ruins, National Park Slovak Paradise, Klastorisko, Slovakia	-

a Redundant closely related clones originating from the same place were removed.

**Table 2:** BLAST results obtained by querying the ITS of *Leptolyngbya* BL0902 with GenBank, and geographical and ecological origins of the hits.

Description^a^	Accession	Query coverage	Score	E value	Identity	Origin of the strain or clone	Reference^b^
Uncultured cyanobacterium isolate DGGE band #38	AY827768	96%	650	0	89%	Lake Klinckenberg, The Netherlands	[66]
*Oscillatoria* sp. strain CCMEE 416	AM398957	87%	477	3.00E-131	82%	Marble point, Antarctica	[67]
*Leptolyngbya antarctica* ANT.ACEV6.1	AY493632	87%	464	2.00E-127	82%	Lake Ace, Vestfold Hills, Antarctica	[62]
Uncultured Antarctic cyanobacterium clone TM2FOCH9	EU852532	87%	462	6.00E-127	81%	Forlidas Pond, Forlidas Valley, Transantarctic Mountains, Antarctica	-
*Leptolyngbya antarctica* TM1FOS73	EU852528	87%	461	2.00E-126	81%	Forlidas Pond, Forlidas Valley, Transantarctic Mountains, Antarctica	-
*Leptolyngbya antarctica* ANT.ACE.1	AY493633	87%	461	2.00E-126	81%	Lake Ace, Vestfold Hills, Antarctica	[62]
Uncultured cyanobacterium clone R8-R60	DQ181762	87%	461	2.00E-126	81%	Lake Rauer 8, Rauer Island, Antarctica	[63]
Uncultured cyanobacterium clone AS-45-2	FJ866611	94%	448	1.00E-122	81%	Submerged sinkhole ecosystem	-
Oscillatoriales cyanobacterium 2Dp86E	GU265558	87%	444	2.00E-121	80%	Hosted by Dynamena pumila L.	[68]
Uncultured Antarctic cyanobacterium clone S334-8	EU032374	87%	443	6.00E-121	82%	Dry Valleys, Antarctica	[69]
Pseudanabaenaceae cyanobacterium DPG1-KK5	EF654067	87%	437	2.00E-119	81%		[61]
*Phormidium* sp. strain AA	AM398947	87%	437	2.00E-119	81%	Desert crust, on sand dunes, Nizzara, Israel	[67]

a Redundant closely related clones originating from the same place were removed.

b A hyphen “”indicates sequences not published in a research article.

**Figure 2:**
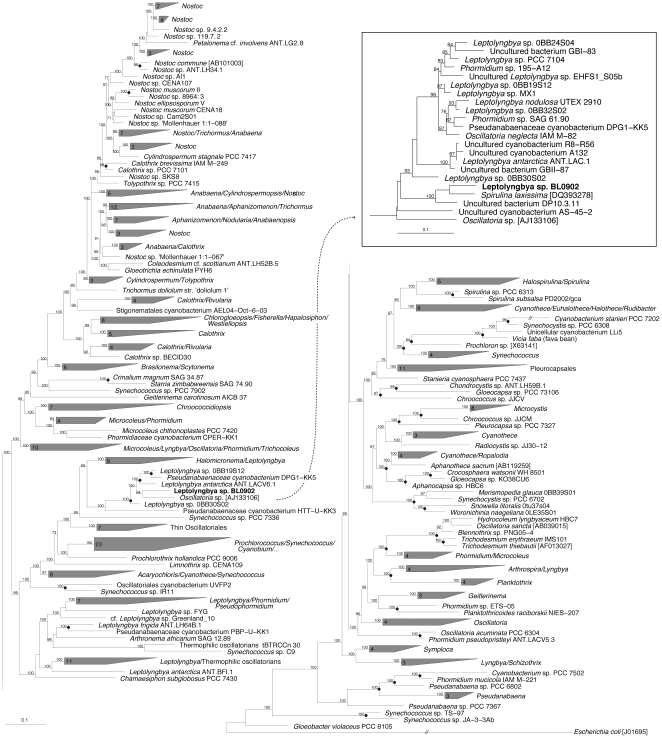
Phylogenetic tree inferred from 16S rRNA gene sequences (*E. coli* positions 110–1440) by maximum likelihood (Likelihood = −58560.000282); branch support values are indicated at the node. Clusters observed using at least 3 construction methods were collapsed or indicated with a black spot at the node. The *E. coli* sequence was used as out-group. The evolutionary distance between two sequences is obtained by adding the lengths of the horizontal branches connecting them and using the scale bars (0.1 mutation per position). The box in the upper right corner displays a subtree comprising sequences not included in the main figure and that share more than 95% similarity with *Leptolyngbya* BL0902.

Nevertheless, *Leptolyngbya* BL0902 belongs to a relatively tight cluster of thin oscillatorians (Fig. 2), mostly assigned to the genus *Leptolyngbya*, including the strains OBB30S02, UTEX 2910, ANT.ACE.1, ANT.ACE.V6.1, 0BB19S12, MXI, 0BB24S04, PCC 7104 (formerly identified as belonging to the LPP group B [24]), UTEX 2910, 0BB32S02, and Kovacik 1999/1. Based on morphological features, “*scillatoria neglecta*”IAM M-82 corresponds more likely to an unknown species of the genus *Leptolyngbya* Anagnostidis & Komárek 1988 (R. Rippka, personal communication). No morphological descriptions of *Spirulina laxissima* SAG 256.80, *Phormidium* sp. 195-A12, *Phormidium* sp. MBIC10025, *Phormidium* sp. SAG 61.90, or *Oscillatoria* sp. [AJ133106] are available. The different taxonomic assignments might be related to either the use of other taxonomic systems or misidentifications.

### Characterization of growth traits

Growth traits including ranges of tolerance for temperature, salinity, pH, light, and urea were determined for *Leptolyngbya* BL0902 and two strains of *Arthrospira*, *A. platensis* BL0909, and *A. maxima* CS-328 (Table 3) as well as 40 other strains (data not shown). *A. platensis* BL0909 had a strict requirement for bicarbonate addition and was unable to grow in BG-11 medium that did not contain bicarbonate. *Leptolyngbya* BL0902 was more versatile with respect to growth media and grew well in both BG-11 and Zarrouk media. All three strains grew well in the 22–40°C temperature range and tolerated up to 0.5 M NaCl, high pH up to 11, and high solar irradiance. Unlike *A. maxima*, *Leptolyngbya* BL0902 was able to tolerate urea at 32 mM, which is commonly used in algal outdoor growth ponds for control of rotifer and amoebae predators.

**Table 3:** Growth traits of *A. maxima*, *A. platensis*, and *Leptolyngbya* BL0902.

Strain	Temperature °C)				NaCl (M)					pH					Light intensity µmol photons m^−2^ s^−1^)				Urea (mM)			
	10	22	30	40	0^a^	0.1	0.25	0.5	1	Std^a^	8	9	10	11	15	125	250	500	0^a^	8	16.7	32
*Arthrospira maxima* CS-328	−	+	+	+	+	+	+	+	−	+	+	+	+	+	+	+	+	+	+	−	−	−
*Arthrospira platensis* BL0909	−	+	+	+	+	+	+	+	−	nd	nd	nd	nd	nd	+	+	+	+/−	nd	nd	nd	nd
*Leptolyngby*a L0902	−	+	+	+	+	+	+	+	−	+	+	+	+	+	+/−	+	+	+	+	+	+	+

a Std, corresponds to BG-11 medium.

+, robust growth; +/−, some growth; −, no significant growth; nd, not determined due to inability of strain BL0909 to grow in BG-11 medium.

### Growth rate and productivity measurements

The doubling time of *Leptolyngbya* BL0902 was measured and compared to *Arthrospira* strains under laboratory and outdoor growth conditions (Table 4). *Leptolyngbya* BL0902 grew faster than both *Arthrospira* species under laboratory conditions. In outdoor open-pond conditions, *Leptolyngbya* sp. BL0902 outperformed *A. maxima* CS-328 and had productivity on par with *A. platensis* BL0909. Importantly, *Leptolyngbya* BL0902 showed good culture stability during 3 months of continuous growth in 1-acre cultivation ponds during the summer 2009 season (Fig. 1e). Additionally, *Leptolyngbya* BL0902 formed long filaments that could be harvested with a vibrating screen similarly to *Arthrospira* spp. (Fig. 1f).

**Table 4:** Doubling time under laboratory conditions and productivity measurements in outdoor ponds.

Strain	Doubling time (h)	Productivity (g m^−2^ day^−1^)
*A. maxima* S-328	32	20
*A. platensis* L0909	24	20–30
*Leptolyngbya* L0902	23	20–25

### Heterotrophic growth

We tested *Leptolyngbya* BL0902 for heterotrophic growth with glycerol and 8 different sugars: glucose, fructose, sucrose, lactose, galactose, arabinose, maltose, and mannose. To prevent growth of potential contaminants, we used a genetically engineered *Leptolyngbya* BL0902 strain expressing the *aadA* gene and supplemented the media with Sp and Sm to a final concentration of 2 µg/ml for each of those antibiotics. The engineered *Leptolyngbya* BL0902 was incubated in the presence of glycerol and each of the 8 sugars at 10 mM final concentration, kept in complete darkness for over 3 weeks or incubated in the light in the presence of the photosynthesis inhibitor DCMU (3-(3,4-dichlorophenyl)-1,1-dimethylurea) at 10 µM final concentration. In both conditions, none of the 8 sugars or glycerol supported growth of *Leptolyngbya* BL0902, demonstrating that *Leptolyngbya* BL0902 cannot grow heterotrophically or photoheterotrophically under these conditions. However, in the presence of glucose, fructose, or sucrose, survival of *Leptolyngbya* BL0902 was improved in the tested conditions.

### Cellular composition and fatty acid profile

**Figure 3:**
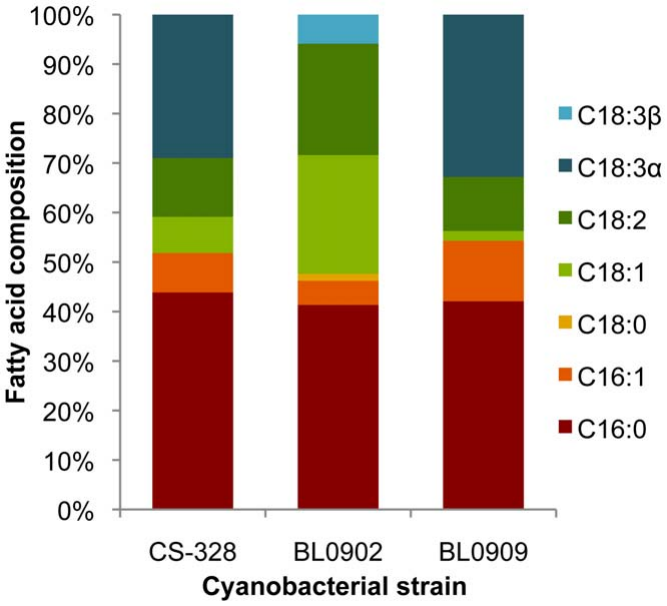
Fatty acid profiles of *A. maxima* CS-328, *Leptolyngbya* BL0902, and *A. platensis* BL0909.

The composition of major cellular components (protein, carbohydrate, fat, ash, fiber, moisture, and fatty acid methyl ester [FAME]) was determined for *A. maxima* CS-328 and *Leptolyngbya* BL0902 (Table 5). Calculated as ash-free dry weight, *Leptolyngbya* BL0902 produced 28.8% FAME compared to 15.6% for *A. maxima* CS-328. Fatty acid profiles are shown in Figure 3. *A. platensis* BL0909 and *A. maxima* CS-328 both contained high levels of tri-unsaturated fatty acids, whereas *Leptolyngbya* BL0902 contained a higher proportion of monounsaturated fatty acids.

**Table 5:** Cellular composition and FAME content as measured by Inventure Chemical.

	*A. maxima* CS-328	*Leptolyngbya* BL0902
moisture	11.8	8.1
protein	55.5	35.4
fat	6.8	5.3
fiber	2.2	3. 7
carbohydrates	9.2	13.3
ash	17.3	34.3
FAME %^a^ (Inventure)	12.9	18.9

a FAME/total dry weight.

### Antibiotic sensitivity

The antibiotic sensitivity of *Leptolyngbya* BL0902 was evaluated for nine antibiotics in BG-11 liquid culture media and on nitrocellulose filters on BG-11 agar plates, which mimics conditions used for genetic conjugations (Table 6). *Leptolyngbya* BL0902 was sensitive to low concentrations of Sp, Sm, Em, and Cm and moderate concentrations of Nm. *Leptolyngbya* BL0902 was somewhat resistant to Km, Gm, and G418 at commonly used concentrations; therefore, these antibiotics could be used to prevent growth of other organisms in laboratory settings. These data provide a panel of antibiotics that could be used as selective markers in genetic manipulations with *Leptolyngbya* BL0902.

**Table 6:** Antibiotic sensitivity of *Leptolyngbya* BL0902.

Antibiotic	×1 final concentration (µg/ml)	Serial dilutions of the antibiotic									
		×1/4		×1/2		×1		×2		×4	
		Liquid	Plate	Liquid	Plate	Liquid	Plate	Liquid	Plate	Liquid	Plate
Chloramphenicol (Cm)	7.5	−	−	−	−	−	−	−	-	−	−
Erythromycin (Em)	20	−	−	−	−	−	−	−	−	−	−
G418	10	nd	+	+	+	nd	+/−	nd	+/−	nd	−
Gentamicin (Gm)	2	+	+	+	+	−	+/−	−	+/−	−	−
Kanamycin (Km)	5	+	+	+	+	+	+	+/−	+/−	−	−
Neomycin (Nm)	25	−	+/−	−	+/−	−	−	−	−	−	−
Spectinomycin (Sm)	2	−	nd	−	nd	−	nd	−	nd	−	nd
Streptomycin (Sp)	2	−	nd	−	nd	−	nd	−	nd	−	nd
Sp+Sm	2 each	−	−	−	−	−	−	−	−	−	−

+, robust growth; +/−, some growth; −, no significant growth; nd, not determined; ×1 final concentration corresponds to an antibiotic concentration commonly used for model strains *Anabaena* sp. PCC 7120 and *Synechococcus elongatus* PCC 7942.

### Conjugal transfer and maintenance of RSF1010-based plasmids

Conjugation from *E. coli* donor cells has been used to introduce DNA into a wide variety of cyanobacteria, and broad-host-range plasmids derived from RSF1010 have been shown to replicate in many strains [18]. To determine whether these methods could be used with *Leptolyngbya* BL0902, we performed biparental matings with *Leptolyngbya* BL0902 and a conjugal *E. coli* donor strain (AM4338) that contained the cargo plasmid pRL1383a, the conjugal plasmid pRL443, and the helper plasmid pRL623. Transconjugant colonies became apparent on selective mating plates after about one week and showed robust growth after transfer to fresh selective plates. Control conjugations without the cargo plasmid never showed any antibiotic resistant colonies.

**Figure 4:**
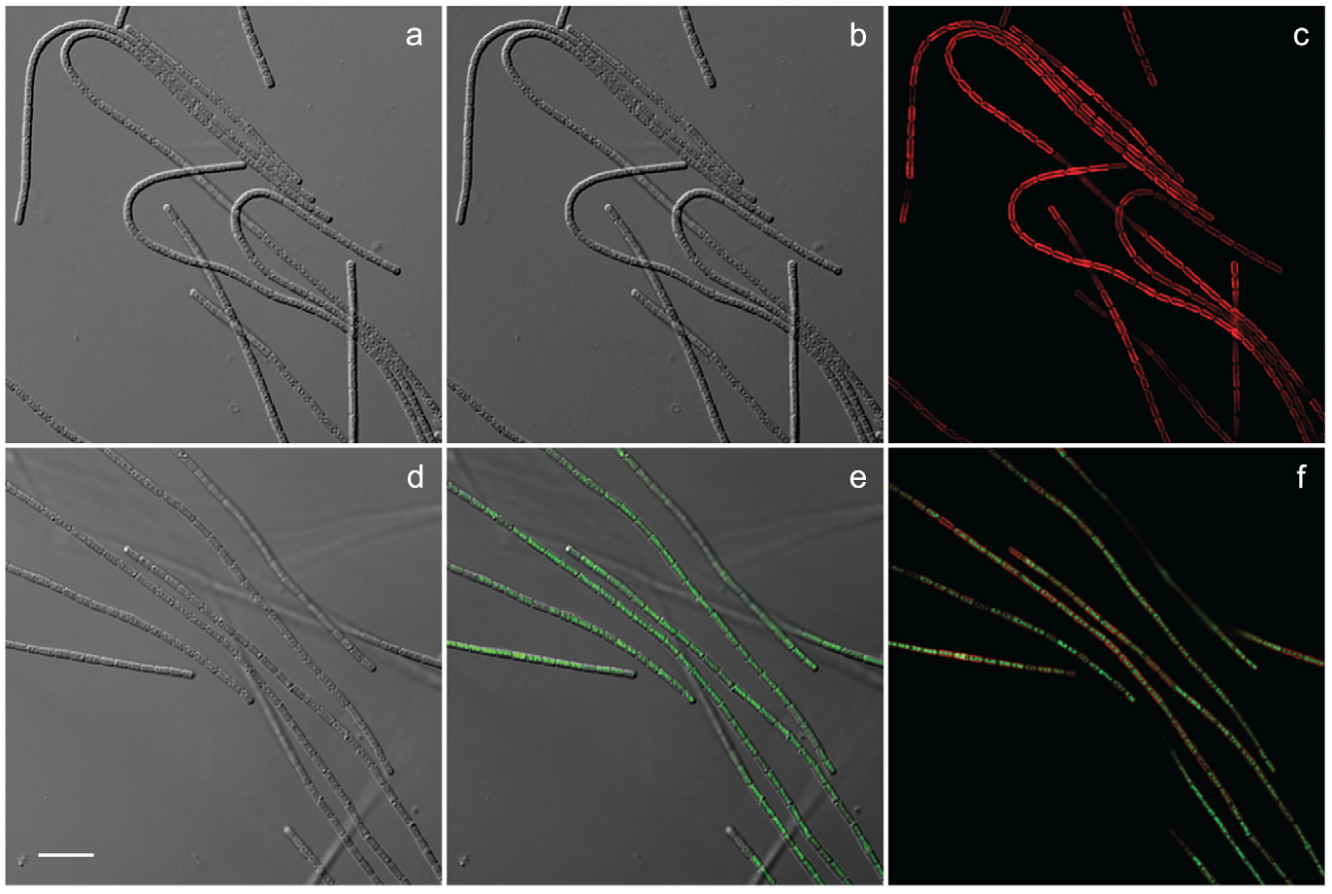
Photomicrographs of wild-type *Leptolyngbya* BL0902 (a, b, and c) and a strain of *Leptolyngbya* BL0902 harboring pAM4418-*yemGFP* expressing the *yemGFP* gene (d, e, and f). (a, d) Differential interference contrast (DIC); (b, e) DIC and green fluorescence; (c, f) Chlorophyll (red) and green fluorescence. Scale bar, 10 µm.

The ability to genetically modify *Leptolyngbya* BL0902 was further demonstrated by the heterologous expression of the *yemGFP* gene. The recombinant plasmid pAM4413 carrying the *yemGFP* gene was electroporated into AM1359, and the resulting strain was conjugated with *Leptolyngbya* BL0902. After one week, isolated transconjugant colonies were restreaked on fresh selective plates, and isolated colonies were then patched to fresh selective plates. Liquid cultures were grown in selective BG-11 medium. Expression of yemGFP was observed by fluorescence microscopy (Fig. 4).

Our initial conjugation experiments were performed with donor strains carrying the helper plasmid pRL623, which carries 3 methylase genes. The methylase genes are required for efficient conjugation into *Anabaena* recipient strains [25]. To assess the necessity of these genes for *Leptolyngbya* BL0902 conjugation, we determined the efficiency of conjugal transfers in biparental and triparental matings with and without the helper plasmid pRL623 and with two different conjugal plasmids: pRL443 and pRK2013 (Table 7). The conjugation protocol was modified as described in the methods to yield more reproducible data for transconjugant colony forming units (CFUs).

**Table 7:** Comparison of conjugal transfer efficiencies in *Leptolyngbya* BL0902 mating experiments.

Exp.	*E. coli* cargo strain	*E. coli* conjugal strain	Mating type	Efficiency (Colonies/CFU)*
1	AM4413 (pAM4413), Sp^r^ Sm^r^	AM4416 (pRK2013), Km^r^	Triparental	0.031±0.011
2	AM4413 (pAM4413), Sp^r^ Sm^r^	AM4415 (pRL443), Ap^r^ Tc^r^	Triparental	0.029±0.011
3	AM4417 (pAM4413, pRL623), Cm^r^ Sp^r^ Sm^r^	AM4416 (pRK2013), Km^r^	Triparental	0.063±0.020
4	AM4417 (pAM4413, pRL623), Cm^r^ Sp^r^ Sm^r^	AM4415 (pRL443), Ap^r^ Tc^r^	Triparental	0.063±0.005
5	AM4414 (pAM4413, pRL623, pRL443), Cm^r^ Sp^r^ Sm^r^ Ap^r^ Tc^r^		Biparental, positive control	0.060±0.026
6	pAM4413 (pAM4413), Sp^r^ Sm^r^		Biparental, negative control	0.000±0.000

*Mean±S.D. (N = 3).

Approximately 3% of potential colony-forming units were transformed by conjugal transfer in bi- or tri-parental matings, which was increased about two-fold when a methylase-expressing helper plasmid was included (Table 7). We did not observe significant differences between bi-parental and tri-parental matings or between the conjugal plasmids pRL443 and pRK2013 (Table 7).

### Transposon mutagenesis

To determine if transposon mutagenesis could be used as a genetic tool with *Leptolyngbya* BL0902, biparental matings were carried out with the *E. coli* strain AM4353, which harbors the Sp^r^ Sm^r^ Em^r^ Tn*5*-692 transposon on the suicide plasmid pRL692, as well as helper and conjugal plasmids (Table 8). Hundreds of Sp^r^ Sm^r^ transconjugant colonies were obtained on mating plates after incubation for a week, and selected Sp^r^ Sm^r^ colonies grew normally on fresh Sp+Sm BG-11 plates and in liquid medium. However, no colonies grew on medium containing Em at concentrations as low as 1.25 µg/ml. BL0902 is very sensitive to Em, and the Tn*5*-692 Em^r^ gene may not be expressed in *Leptolyngbya* BL0902. A repetition of the transposon-tagging experiment produced similar results.

**Table 8:** Plasmids and strains.

Strain or plasmid	Derivation and/or relevant characteristic	Source or reference
Plasmids		
pAM2255	Expression vector carrying a *trc* (IPTG inducible) promoter, Ap^r^	H. Iwasaki, [70]
pAM4413	pRL1383a vector carrying the *yemGFP* open reading frame downstream of *trc* constitutive promoter, Sp^r^ Sm^r^	This study
pAM4418	Conjugal destination vector pRL1383a carrying an IPTG inducible *trc* promoter upstream of a Gateway recombination cassette, Sp^r^ Sm^r^ Cm^r^	This study
pAM4418-yemGFP	Expression plasmid resulting from a Gateway LR recombination reaction of pENTR-SD-yemGFP and pAM4418, Sp^r^ Sm^r^	This study
pDEST_M3	Gateway destination vector optimized for *Synechococcus elongatus* PCC 7942 and carries *lacI* ^q^ and *trc* promoter upstream of a recombination cassette. Ap^r^ Km^r^ Cm^r^	This study
pENTR-SD/D-TOPO	Gateway entry vector with Shine-Dalgarno sequence; Km^r^	Invitrogen
pENTR-SD-yemGFP	pENTR-SD/D-TOPO carrying the *yemGFP* open reading frame, Km^r^	This study
pEXP_1ax-yemGFP	Expression vector carrying *yemGFP* downstream of an IPTG inducible promoter, Sp^r^ Sm^r^ Cm^r^	This study
pJS151	Expression plasmid containing yemGFP, a GFP allele with F64L, S65T, and A206K mutations and that has been codon-optimized for expression in yeast. The first two mutations correspond to mut1GFP [71], and the third mutation interferes with GFP dimerization [72]	J. Hasty
pRK2013	Conjugal plasmid, derivative of RK2, Km^r^	J. Meeks, [34], [53]
pRL443	Conjugal plasmid, Km^s^ derivative of RP4, Ap^r^ Tc^r^	[25]
pRL623	Helper plasmid carrying Mob_ColK_ and methylase genes M.AvaI, M.Eco47II, M.EcoT22I, Cm^r^	[25]
pRL692	Transposon mutagenesis plasmid carrying the mobile element Tn*5*-692 that contains a pMB1 oriV, Sp^r^ Sm^r^ Em^r^	(GenBank Accession No. AF424805) [36]
pRL1383a	Mobilizable, broad host range plasmid derived from RSF1010, Sp^r^ Sm^r^	(GenBank Accession No. AF403426) [54]
		
*E. coli* strains		
DH5α	Cloning host	Gibco BRL
DH10B	Cloning host	Gibco BRL
One Shot ccdB Survival™ (T1R)	Cloning host	Invitrogen
One Shot TOP10	Cloning host	Invitrogen
AM1358	DH10B harboring pRL623, Cm^r^	[52]
AM1359	DH10B harboring pRL623 and pRL443, Cm^r^ Ap^r^ Tc^r^	[73]
AM4338	AM1359 harboring pRL1383a, Cm^r^ Ap^r^ Tc^r^ Sp^r^ Sm^r^	This study
AM4353	AM1359 harboring pRL692, Cm^r^ Ap^r^ Tc^r^ Sp^r^ Sm^r^ Em^r^	This study
AM4389	DH10B harboring pENTR-SD-yemGFP, Km^r^	This study
AM4413	DH10B harboring pAM4413, Sp^r^ Sm^r^	This study
AM4414	AM1359 harboring pAM4413, Cm^r^ Ap^r^ Tc^r^ Sp^r^ Sm^r^	This study
AM4415	DH10B harboring pRL443, Ap^r^ Tc^r^	This study
AM4416	DH10B harboring pRK2013, Km^r^	This study
AM4417	AM1358 harboring pAM4413, Cm^r^ Sp^r^ Sm^r^	This study
AM4503	One Shot ccdB Survival™ harboring pDEST_M3, Km^r^ Ap^r^ Cm^r^	This study
AM4517	DH5α harboring pAM4418-yemGFP, Sp^r^ Sm^r^	This study
		
Cyanobacterial strains		
BL0902	Wild type	This study
BL0909	Wild type	This study
CS-328	Wild type	D. Bryant
PCC 7120	Wild type	Laboratory collection
PCC 7942 (AMC006)	Wild type	Laboratory collection

**Figure 5:**
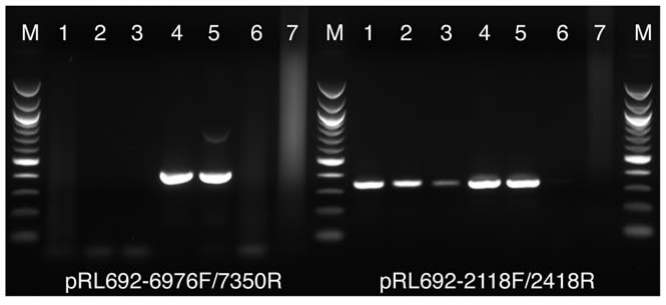
PCR assays showing integration of the Tn*5*-692 transposon into the chromosome of *Leptolyngbya* BL0902. (lanes 1, 2, 3) Transconjugant *Leptolyngbya* BL0902 clones, (lane 4) *E. coli* strain AM4353 harboring pRL692, (lane 5) pRL692 DNA, (lane 6) WT *Leptolyngbya* BL0902, (lane 7) no template DNA, (lane M) 100-bp ladder size marker. Primer pairs used to amplify the plasmid backbone (left) and the Tn*5*-692 transposon (right) are shown at the bottom.

The integration of the Tn*5*-692 transposon into the *Leptolyngbya* BL0902 chromosome was confirmed by a set of PCR assays carried out on three putative transconjugant clones. The clones were grown in BG-11 liquid culture, which resulted in the loss of all viable donor *E. coli* cells. The absence of *E. coli* was confirmed by a lack of colony formation when transconjugant cyanobacterial samples were inoculated on BG-11 plates supplemented with 0.04% (wt/vol) glucose and 5% (vol/vol) LB broth and incubated in the dark at 30°C, or on LB plates incubated at 37°C. Two pairs of primers were used for the PCR assays. The primer pair pRL692-6976F/7350R (Table 9) amplifies a 421-bp fragment within the origin of transfer (OriT) of the plasmid backbone from position 6953 to position 7373 of pRL692. The primer pair pRL692-2118F/2418R amplifies a 347-bp fragment within the transposon Tn*5*-692 from position 2095 to position 2441 of pRL692. The OriT primer pair produced PCR products in the positive-control samples only (Fig. 5, lanes 4 and 5), indicating the absence of the suicide plasmid in any of the three transconjugants and confirming the loss of all *E. coli* cells. The Tn*5*-692 primer pair produced PCR products from all three transconjugant strains and the positive controls, but not from WT *Leptolyngbya* BL0902. These data show that the Tn*5*-692 transposon can be used for transposon tagging in *Leptolyngbya* BL0902.

**Table 9:** Primers.

Primer name	Sequence
lacIq_F (EcoRI)	5′-GAGTCAAGAATTCGTGGTGAATGTGAAACC-3′
pRL692-2118F	5′-TACCGATACAACTACTGGTGAGGA-3′
pRL692-2418R	5′TATCTCAGCGATCTGTCTATTTCG-3′
pRL692-6976F	5′-GTACTTACAGCTCGAAGTGCCTCT-3′
pRL692-7350R	5′-CTATCAAGGTGTACTGCCTTCCAG-3′
rrnB_R (AvrII)	5′-AAATAACCTAGGGAGTTTGTAGAAACGCAAAAAG-3′
yemGFP_F	5′-CACCATGTCTAAAGGTGAAGAATTATTCACTG-3′
yemGFP_R	5′-TTATTTGTACAATTCATCCATACCAT-3′

### Construction and testing of the pAM4418 expression vector

**Figure 6:**
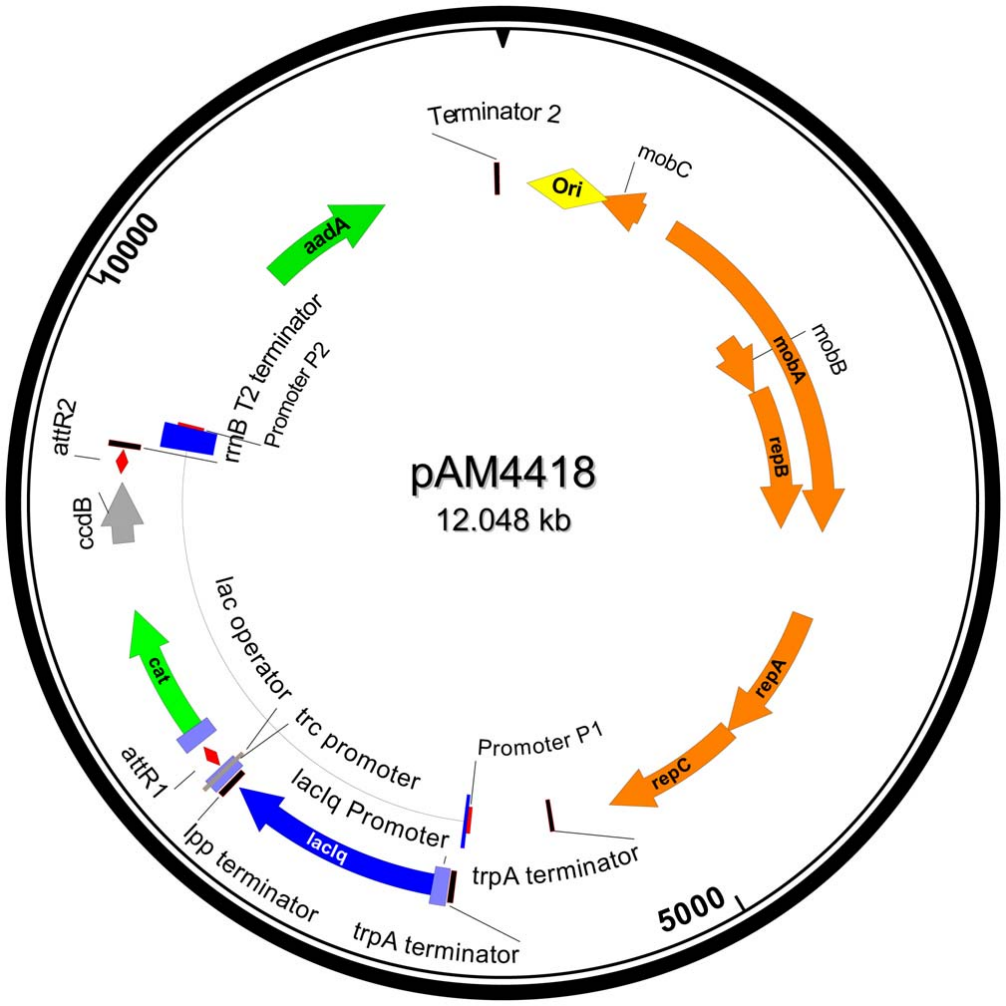
Map of the engineered shuttle plasmid pAM4418 carrying *trpA* terminator, *lacI^q^* promoter and gene, terminator from the *E. coli lpp* gene, *trc* promoter, Gateway recombination cassette, T2 terminator from *rrnB*, and backbone of pRL1383a. Map drawn with SeqBuilder (Lasergene 8, DNASTAR).

To facilitate the ability to introduce and express genes or noncoding and antisense RNAs in *Leptolyngbya* BL0902 and other cyanobacterial strains, we constructed plasmid pAM4418 based on the conjugal vector pRL1383a (Fig. 6). pAM4418 contains an *E. coli lacI^q^* gene and the inducible *trc* promoter upstream of a Gateway recombination cassette. Genes of interest that are cloned in a pENTR vector can be introduced into pAM4418 by an LR recombination reaction. We monitored the expression of yemGFP as fluorescence emission intensity in *Leptolyngbya* BL0902 harboring pAM4418-*yemGFP* for two days following induction with IPTG. The reporter was constitutively expressed at moderately high levels, but there was no significant increase in yemGFP fluorescence intensity with IPTG addition at final concentrations ranging from 0.1 to 10 mM. We conclude that the *trc* promoter functions well in *Leptolyngbya* BL0902, but that either the *lacI^q^* gene is not expressed or the LacI protein fails to repress expression from the *trc* promoter on pAM4418.

## Discussion

*Leptolyngbya* BL0902 provides a new experimental model for cyanobacterial research that is focused on the goal of outdoor commercial production. Its growth traits related to harvestability, temperature range, and tolerance of high salt, pH, and light, paired with facile genetic manipulation, make *Leptolyngbya* BL0902 a potential commercial production platform strain. *Leptolyngbya* BL0902 growth rates in the laboratory and in outdoor ponds were similar to those of *Arthrospira* spp. that are currently grown at commercial scales, and large-scale outdoor pond cultures showed excellent stability during 3 months of growth in the summer of 2009. This is noteworthy because 13 out of 15 tested strains failed attempts to scale up to 1-acre growth ponds (unpublished observations).

Morphology and molecular data (16S rRNA and ITS gene sequences) place *Leptolyngbya* BL0902 as a novel isolate of this genus, within a cluster of thin oscillatorians isolated from a variety of biotopes and locations, which suggests a high resilience and competitiveness in a range of environmental conditions. The *Leptolyngbya* genus is heterogeneous and polyphyletic with a high genotypic diversity hidden behind a simple morphology. Specimens have been reported from hypersaline, marine, and freshwater habitats ranging from Antarctic lakes to hot springs. Most would have originally been identified as species of *Lyngbya* Agardh 1824, *Phormidium* Kutzing 1843, *Plectonema* Thuret 1875, or *Oscillatoria* Vaucher 1803, and were grouped under the name LPP [24], [26]. This group was later revised to form a new genus, *Leptolyngbya* [27].

*Leptolyngbya* BL0902 accumulated higher FAME content and a higher proportion of mono-unsaturated fatty acids, preferable for a biodiesel feedstock, than two strains of *Arthrospira* spp.; the latter have high levels of tri-unsaturated fatty acids, preferable for nutritional applications but not desirable for fuel applications due to low oxidative stability. FAME recovery by a proprietary direct conversion process (Inventure Chemical, Inc.) for *Leptolyngbya* BL0902 and other cyanobacterial strains was significantly higher than has been reported by standard Bligh-Dyer extraction for cyanobacterial strains [28]. Further improvement of the *Leptolyngbya* BL0902 fatty acid profile may be achieved by overexpressing the native or a heterologous Δ-9 acyl-lipid desaturase to increase the proportion of monounsaturated fatty acids.

Microalgal industrial production strains will need to be genetically manipulable. At least thirty-three different strains of cyanobacteria have been transformed, and a variety of genetic tools have become available since the unicellular cyanobacterium *S. elongatus* PCC 7942 (formerly *Anacystis nidulans* R2) was transformed four decades ago [18], [29]. While transformation and electroporation are used for some strains, including a few naturally transformable cyanobacteria [16], conjugation, first shown in *Anabaena* sp. strain PCC 7120 [30], is generally the most successful and efficient method for gene transfer into cyanobacteria [31]. Conjugal plasmids derived from the related IncPα plasmids RP4 and RK2 [32], including pRL443 and pRK2013, have been used to mediate transfer of engineered cargo plasmids into several strains [16]. We demonstrated that broad host range plasmid vectors based on RSF1010 can be efficiently transferred to and stably maintained in *Leptolyngbya* BL0902. Previous studies have found that pRK2013 and its Km^s^ derivative pRK2073 promote increased conjugal transfer efficiencies in 3 strains of *Chroococcidiopsis* species and *Nostoc punctiforme* ATCC 29133 [33], [34], but pRL443 and pRK2013 performed similarly in our study.

The presence of the helper plasmid pRL623, which carries three restriction methylase genes and is necessary to overcome restriction barriers in *Anabaena* sp. strain PCC 7120 [25], increased conjugation efficiency in *Leptolyngbya* BL0902 by only two-fold. Restriction systems usually result in order-of-magnitude differences in conjugation efficiencies, but our results indicated little protective role for the methyltransferases carried by pRL623. Therefore, restriction systems do not appear to pose a significant barrier to genetic manipulation of *Leptolyngbya* BL0902, and the conjugation efficiency with or without a helper plasmid is on par with the efficiency reported for *Anabaena* sp. strain PCC 7120 [25]. Triparental and biparental matings involving the same set of plasmids performed similarly. Triparental matings, in which the conjugal and mobilizable cargo plasmids are not in the same cell at the start of mating [35] allow the use of plasmids from the same incompatibility group or that carry the same selectable markers.

Transposon mutagenesis is a powerful tool for gene discovery. As in the heterocystous and unicellular cyanobacterial strains *Anabaena variabilis* ATCC 29413 and *S. elongatus* [36], Tn*5*-692 is capable of transposition in *Leptolyngbya* BL0902. The high frequency of stable antibiotic-resistant colonies indicate that transposon mutagenesis will be a useful method for identifying new genes in *Leptolyngbya* BL0902 that are involved in traits related to large-scale growth, such as growth rate in open ponds and resistance to predators and pathogens. Gene discovery in *Leptolyngbya* BL0902 will be enhanced by the availability of a complete genome sequence, which is underway. Application of these genetic tools can lead to rapid strain modifications for improved growth properties, and the production of biomass and desired molecules such as renewable biofuels. *Leptolyngbya* species are not generally known to produce toxins, however there is a report of a toxin-related gene in a marine *Leptolyngbya* strain [37]. Identification and targeted inactivation of toxin genes would be another obvious goal for engineered strain improvement. Our work also provides a basis for developing gene transfer methods and genetic engineering tools for new strains of cyanobacteria that possess desirable characteristics for growth in a variety of different conditions and geographic locations.

## Materials and Methods

### Ethics Statement

This research involved field studies of algal strains grown in outdoor ponds at Carbon Capture Corporation' Algae Research Center in Imperial Valley, California, which was leased by Biolight Harvesting, Inc. during the field studies described in this work. No specific permits were required for the described field studies, which were performed at a leased commercial facility, and which did not involve endangered or protected species.

### Strain isolation

Plasmids and strains related to this work are listed in Table 8. *Leptolyngbya* BL0902 was isolated from an open pond at the Carbon Capture Corporation Algae Research Center in Imperial Valley, CA. A sample of the pond water was serially diluted and incubated in 96 well plates at 30°C under 100-µmol photons m^−2^ s^−1^ constant light in BG-11 or Zarrouk medium. Unialgal wells were subcultured and, following visual examination, the best-growing non-redundant cultures were chosen as representatives of the strains present in the open ponds. *Leptolyngbya* BL0902 was one of the isolates. An axenic culture of *Leptolyngbya* BL0902 was obtained by picking isolated and “lean”filaments under a dissecting microscope and repeatedly streaking on agar-solidified BG-11 and BG-11 supplemented with 0.04% (wt/vol) glucose and 5% (vol/vol) LB (Lennox broth) (BG-11 Omni medium) followed by repeated serial dilution of the culture in liquid BG-11. To verify that the strain was axenic, cloned isolates were inoculated into 4 different solid and liquid media: (1) BG-11 Omni medium, (2) BG-11 supplemented with 0.01% (wt/vol) glucose, yeast extract (Difco), and Bacto-Peptone (Difco), (3) Gram-negative broth (GNB) medium (Difco), and (4) LB. Solid and liquid cultures were incubated at room temperature, 30°C, and 37°C in the dark. If no growth of heterotrophic bacteria was observed under any of the conditions after incubation for 1 month, the cultures were judged axenic. The isolates were also checked for contamination by differential interference contrast (DIC) and fluorescence microscopy after being stained with DAPI (4′,6-diamidino-2-phenylindole) at 10 µg ml^−1^. Strains were stored at −80°C in medium supplemented with 8% DMSO.

### Microscopy, morphological description, and identification

Bright field, DIC, and phase contrast photomicroscopy were carried out with a Zeiss Axioskop microscope equipped with Plan-Neofluar 40x/0.75 and 100x/1.30 objectives and a SPOT RT3 25.4 2 Mp Slider camera. DIC and fluorescence microscopy were carried out and images were captured on a Delta Vision (Applied Precision, Inc.) microscope system composed of an Olympus IX71 inverted microscope equipped with an Olympus UPlanSApo 100×/1.40 objective and a CoolSNAP HQ2/ICX285 camera. Tetramethylrhodamine isothiocyanate (TRITC) filters (S555/25 excitation and S630/60 emission) were used to image autofluorescence of photosynthetic pigments, and GFP filters (S484/16 excitation and S515/30 emission) were used to image GFP fluorescence. Image acquisition, deconvolution, and analysis (cell measurements) were performed using Resolve3D softWoRx-Acquire (Version 4.0.0) and Adobe Photoshop CS4.

Morphological description and identification were based on the taxonomic work of Komárek and Anagnostidis [38].

### Molecular identification

PCR amplification of the 16S rRNA gene plus the internal transcribed spacer (ITS) between the 16S rRNA gene and the 23S rRNA gene was carried out from an isolated colony of *Leptolyngbya* BL0902 using the primer pair 16S27F/23S30R as described previously [39]. Sequencing was carried out by GENEWIZ (La Jolla, CA, USA) using the primers: 16S27F, 16S378F, 16S1490R, and 23S30R [39]. Base calling and sequence assemblies were made using the software package Phred/Phrap and Consed [40]–[42].

The 16S rRNA gene sequence (*E. coli* positions: 101–1449) and the ITS of *Leptolyngbya* BL0902 were initially analyzed by similarity search using the basic local alignment search tool (BLAST) software. The 16S rRNA gene sequence of *Leptolyngbya* BL0902 was added to the database of the ARB software package [43] and aligned with the reference alignment ‘ILVA SSU Ref 100’[44]. For further analyses, 328 sequences covering the *E. coli* positions 110–1440 were chosen with the software mothur [45] as one representative sequence per OTU (operational taxonomic unit), which was defined as a group of sequences sharing at least 97.5% identity. Ambiguously aligned positions were deleted from the alignment using Gblocks 0.91b [46] with settings that allowed the most relaxed selection of blocks. Phylogenetic trees were constructed using four methods: (1) The Maximum Likelihood of PHYML [47] using a SH-like branch support and based on a GTR+I+G model using 4 categories of substitution rate; the GTR+I+G model was determined to be the most appropriate to our dataset according to the Perl script MrAIC (version 1.4.3, Evolutionary Biology Centre, Uppsala University, Sweden [http://www.abc.se/~nylander/mraic/mraic.html]); the proportion of invariant sites and gamma distribution parameter were estimated by PHYML from the dataset. (2) The Wagner parsimony of DNAPARS as implemented in PHYLIP 3.69 [48] with the jumble option set to 10 and global rearrangements that involved the construction of 4800 trees. (3) The Bayesian Markov Chain Monte Carlo method as implemented in BEAST [49] based on a GTR+I+G model using 4 categories of substitution rate (ChainLength = 1.10^6^, LogEvery = 100). (4) The Neighbour joining method on a Jukes and Cantor distances matrix as implemented in PHYLIP with a bootstrap analysis involving the construction of 1000 trees. Related sequences sharing more than 95% similarity with *Leptolyngbya* BL0902 not included in the above-mentioned selection were incorporated in Figure 2 as a subtree (boxed) built according to the first method afore mentioned.

### Characterization of growth traits

Ranges of tolerances for temperature, salinity, pH, light intensity, and urea concentration were determined for *Leptolyngbya* BL0902 and compared with 40 other cyanobacterial strains (data not shown) including two strains of *Arthrospira*, *A. platensis* BL0909 and *A. maxima* CS-328. Traits were assessed in 6- or 24-well plates containing liquid BG-11 or BG-11 supplemented with 20 mM NaHCO_3_ for *Arthrospira* spp. Unless temperature or light intensity was being investigated, cultures were maintained at 30°C under continuous light with an intensity of 125 µmol photons m^−2^ s^−1^ as measured with a QSL-100 Quantum light meter (Biospherical Instruments, Inc.). The temperature effect on growth was evaluated at 10°C, 22°C, 30°C, and 40°C, and the effect of light intensity was evaluated at 15, 125, 250, and 500 µmol photons m^−2^ s^−1^. To assess growth at various NaCl concentrations, BG-11 medium containing 20 mM HEPES (pH 8.0) was adjusted to final concentrations of 0.1, 0.25, 0.5, 1, and 2 M NaCl. The influence of pH on growth was investigated with culture media adjusted to pH 8.0 with 10 mM HEPES, pH 9.0 and 10.0 with 10 mM CHES, and pH 11.0 with 10 mM CAPS. Unbuffered BG-11 (pH∼7.5) was used as a control. Tolerance to urea was determined by addition of urea to final concentrations of 8, 16.7, 32, 64, and 100 mM. All experiments included control BG-11 samples. Cultures were incubated for 2 weeks except for growth at 10°C, for which plates were incubated for up to one month. Growth was determined by visual assessment.

The doubling time of *Leptolyngbya* BL0902 was measured and compared to *A. platensis* BL0909 and *A. maxima* CS-328 under both laboratory and outdoor growth conditions. Laboratory cultures were grown in 100 ml Zarrouk medium in 250 ml flasks on orbital shakers illuminated with 100 µmol photons m^−2^ s^−1^ in 12∶12 h light:dark and 35∶25°C temperature cycles. Optical densities at 750 nm were used to determine doubling times. *Leptolyngbya* BL0902 cultures were also grown in outdoor raceway ponds of an algae farm located in the Imperial Valley, CA, USA for more than 3 months continuously during the summer of 2009. The biggest ponds were about 1.2 acres and 15 cm deep, with a paddle wheel-driven flow speed of about 9 m/min (Fig. 1e). Daily average air temperatures in the Imperial Valley during the summer of 2009 (June 1 - September 30) were between 25.6°C and 40.8°C, with the lowest and highest temperatures being 16.6°C and 46.6°C, respectively. During this period, there was between 12 and 14 h of daylight with no significant precipitation and little to no cloud cover. Cyanobacterial filaments were harvested using a sloped 50 µm vibrating screen (Fig. 1f). The slurry from the screen was rinsed with fresh water, dewatered using the vibrating screen, and then spread on a cement slab to dry for two days.

### Heterotrophic growth

*Leptolyngbya* BL0902 was tested for heterotrophic growth with glycerol and 8 different sugars: glucose, fructose, sucrose, lactose, galactose, arabinose, maltose, and mannose. To suppress growth of bacterial contaminants, these experiments were performed with a *Leptolyngbya* BL0902 strain containing pRL1383a, and 2 µg/ml each of spectinomycin and streptomycin were added to the growth media. The strain was incubated in the presence of glycerol or each of the 8 sugars at 10 mM final concentration and either kept in complete darkness for over 3 weeks or incubated in the light in the presence of the photosynthesis inhibitor DCMU (3-(3,4-dichlorophenyl)-1,1-dimethylurea) at 10 µM final concentration.

### Cellular composition and fatty acid profile

Proportions of the major cellular components including protein, carbohydrate, fat, ash, fiber, and moisture were determined by New Jersey Feed Lab. Inventure Chemical determined the percentage of fatty acid methyl ester (FAME) using 100 g dried samples collected from an outdoor open pond.

To determine fatty acid profiles, lipids were isolated from cell pellets using a modified Bligh-Dyer extraction [50] followed by transesterification with sodium methoxide, and GC-MS analysis. Samples (5 ml) of an exponentially growing culture (OD_750_ ∼0.8) were collected by centrifugation and resuspended in 0.8 ml of H_2_O. 3 ml CHCl_3_:MeOH (1∶2) was added and the vials were vortexed for 1 min. After 1 h incubation at 60°C, 1 ml of CHCl_3_ was added and the vials were vortexed for 1 min. Then, 1 ml of H_2_O was added, the vials were vortexed for 1 min and briefly centrifuged. The lower layer was recovered into a fresh vial and solvent was removed under a stream of nitrogen. 1 ml of 0.5 M sodium methoxide in MeOH was used to resuspend the dried crude lipid and the reaction was incubated for 30 min at room temperature. The reaction was quenched with 1 ml of H_2_O and the resulting methyl esters were recovered into 2 ml of hexane by vortexing for 1 min. The hexane layer was clarified by centrifugation and sampled for GCMS analysis. The extracts were analyzed on an Agilent 6890N GC equipped with a DB-FFAP column (30 m length, 0.25 mm ID, and 0.50 µm film thickness) coupled to a 5973 inert mass selective detector (Agilent Technologies, Inc.). Helium was used as the carrier gas with a flow rate of 1.2 ml/min, and 1 µl was injected into the column with a 50∶1 split ratio. The column temperature was held at 100°C for 5 min and then ramped at 10°C/min to 250°C and held for 10 min. The total run time was 30 min. Identification of the fatty acids was based on retention times and fragmentation patterns of standards.

### Antibiotic sensitivity evaluation

The antibiotic sensitivity of *Leptolyngbya* BL0902 was evaluated against a panel of antibiotics in BG-11 liquid culture media and on 25 mm nitrocellulose filters laid on BG-11 agar. The tested antibiotic concentrations were ¼, ½, 1, 2, and 4 times the concentrations commonly used in our laboratory for the selection of recombinant cyanobacterial strains: 5 µg/ml kanamycin (Km), 2 µg/ml gentamicin (Gm), 20 µg/ml erythromycin (Em), 7.5 µg/ml chloramphenicol (Cm), 25 µg/ml neomycin (Nm), and 10 µg/ml G418. 2 µg/ml each of streptomycin (Sp) and spectinomycin (Sm) were used together for Sp^r^/Sm^r^ strains to limit the appearance of spontaneous resistant mutants.

### Mating and conjugal transfer of plasmid DNA

Transformations of *Leptolyngbya* BL0902 through biparental and triparental conjugations followed published protocols [25], [51], [52] with minor modifications. Our standard biparental matings involved the cyanobacterial strain *Leptolyngbya* BL0902 and an *E. coli* strain (DH10B) that harbored the following three plasmids: (i) the conjugal plasmid pRL443, an Ap^r^ Tc^r^ Km^s^ derivative of RP4 [51], or pRK2013, a Km^r^ plasmid containing the transfer genes of RK2 cloned onto a ColE1 replicon [34], [53], (ii) the “elper”plasmid pRL623, which carries the gene for Mob_ColK_ and methylase genes encoding M.AvaI, M.Eco47II, whose product methylates AvaII sites, and M.EcoT22I, an isoschizomer of M.AvaIII [25], and (iii) the cargo plasmid pRL1383a, pAM4413, or pRL692. Plasmid pRL1383a (GenBank Accession No. AF403426) is a Sp^r^ Sm^r^ derivative of RSF1010 [54] and pRL692 (GenBank Accession No. AF424805) carries the Sp^r^/Sm^r^ and Em^r^ mobile element Tn*5*-692 [36]. Triparental matings involved the strain BL0902 and two *E. coli* strains: a cargo strain carrying the cargo plasmid with or without a helper plasmid and a conjugal strain carrying a conjugal plasmid.

*E. coli* strains were grown in 3 ml LB with the appropriate antibiotic(s) and incubated at 37°C overnight. Cells were harvested from 2 ml of each *E. coli* culture by centrifugation and resuspended in 2 ml fresh LB. This step was repeated twice to wash the cells. After the third centrifugation, the cells were resuspended in 200 µl BG-11. Five milliliters of a growing *Leptolyngbya* BL0902 culture were harvested by centrifugation at low speed (4000 × g) and resuspended in 1 ml BG-11. The filaments were then fragmented in a water bath sonicator for 5 to 15 min so that more than half of the filaments were shorter than 5 cells. Fragmentation of filaments is not essential for efficient conjugation but is required for quantitative experiments. The cyanobacterial cells were collected by centrifugation for 2 min and resuspended in 1 ml BG-11. The cargo strain, the conjugal strain (for triparental mating), and *Leptolyngbya* BL0902 were combined, pelleted by centrifugation, and finally resuspended in 200 µl BG-11. The conjugation mixture was incubated for about 1 h in low light at 30°C; however this incubation step may be unnecessary and is possibly even detrimental to conjugation efficiency. The cells were collected by centrifugation, resuspended with a small volume of BG-11, and then spread on sterile nitrocellulose filters laid on BG−11+5% (vol/vol) LB agar plates (mating plates). The mating plates were incubated without antibiotic selection for 18 to 24 h in low light at 30°C, and then the filters were transferred to BG-11 agar with 2 µg/ml each Sp and Sm. After incubation for 6 to 8 days, isolated transconjugant colonies were patched on fresh selective BG-11 plates. Finally, cyanobacterial cells scraped from grown patches were transferred to 100 ml of selective liquid BG-11 in 250 ml flasks and grown at 27–30°C and 100 µmol photons m^−2^ s^−1^.

For experiments to test conjugation efficiency the protocol was modified slightly to allow better reproducibility for comparisons between experiments. The *E. coli* strains were grown overnight in 3 ml LB containing appropriate antibiotic(s), and 2 ml of culture were transferred to 25 or 50 ml LB plus antibiotic(s) and grown for a few hours to an OD_600_ of 0.6 to 0.8. Each culture was then diluted to an OD_600_ of 0.6, and for each mating, 2 ml samples were washed twice with LB medium and resuspended in 0.2 ml BG-11. For triparental matings, 2 ml of each of the two *E. coli* strains were combined before resuspension in 0.2 ml BG-11. For the recipient cells, a 100 ml BG-11 culture of *Leptolyngbya* BL0902 was grown to an OD_750_ of 0.7. Four aliquots of the culture (approximately 25 ml each) were transferred to 50 ml conical centrifugation tubes, and the filaments were fragmented by sonication using a needle probe with ten 5-second pulses separated by 5-second pauses at a power setting of 20%, which resulted in short filaments of which about half were 3 or fewer cells in length. The fragmented filaments were collected by centrifugation at 4000 × g for 10 min and resuspended in 20 ml BG-11. Each mating contained 1 ml of *Leptolyngbya* BL0902 concentrated cells and 0.2 ml of concentrated *E. coli* cells. For each mating, 7.5 and 30 µl of the conjugation mixture, corresponding to about 3×10^6^ and 1×10^7^ short filaments (estimated microscopically with a hemocytometer), respectively, were adjusted to 150 µl with BG-11, and the cells were evenly spread on 90 mm nitrocellulose filters lying on mating plates using about 2 g of sterilized glass beads (2 to 4 mm diameter). To determine the total number of CFU in each conjugation mixture, 1 µl was serially diluted to 10^−4^ and 10^−5^, and 150 µl of each dilution, corresponding to about 6×10^2^ and 6×10^3^ short filaments per ml, respectively, was plated and grown in parallel with the conjugation experiments.

### Construction of recombinant plasmids based on the pRL1383a backbone

The pRL1383a backbone includes the following modules: multiple cloning site, SP6 promoter from pBAC108L (GenBank U51114), *aadA* promoter and gene conferring Sp^r^ Sm^r^, *rrnC* terminator from Lorist6 (GenBank X98450), origin of replication, *mob* genes, *rep* genes, *trpA* terminator from Lorist6 (GenBank X98450), and T7 promoter from pBAC108L (GenBank U51114).

To construct pAM4413, a PCR fragment that included a *lacI^q^* gene with an S289L mutation (pAM2255) and a *trc* promoter with an R80I mutation (pAM2255), a *yemGFP* (yeast-enhanced monomeric green fluorescent protein) gene with F64L, S65T, and A206K mutations, and a *rrnB* transcriptional terminator (pAM2255) was amplified from the pEXP_1ax-yemGFP plasmid with the primers laclq_F and rrnB_R (Table 9) carrying the restriction sites *Eco*RI and *Avr*II, respectively. The PCR fragment was gel purified and ligated into pRL1383a to replace the fragment between the *Eco*RI and *Avr*II restriction sites.

The destination vector pAM4418 was constructed by ligation of a pDEST_M3 fragment and pRL1383a. The pDEST_M3 fragment included the following modules: *trp*A terminator [55], *lacI^q^* promoter with −35 to +1 region replaced by the conII synthetic promoter [56], *lacI^q^* gene (synthetic ORF codon optimized for *Synechococcus elongatus* PCC 7492), *Ipp* transcriptional terminator [57], *trc* promoter (ends defined by the overlap between pTrcHis2-A for the 5′ end and pAM2255 for the 3′ end), Gateway cassette reading frame A comprising *cat* (chloramphenicol resistance) and *ccdB* (DNA gyrase toxin [58]) genes flanked by attR1 and attR2 recombination sites (Invitrogen), and the *rrnB* T2 terminator [59]. The pDEST_M3 fragment was isolated with NaeI and HindIII, treated with the T4 polymerase to generate blunt ends, and gel purified by electrophoresis. pRL1383a was linearized with HincII and dephosphorylated with CIP to prevent self-ligation. The ligation was transformed into One Shot *ccdB* Survival T1 Phage-Resistant (T1^R^) chemically competent *E. coli* (Invitrogen).

To construct pAM4418-based expression plasmids, the gene of interest needs to be amplified by PCR using a forward primer carrying a CACC motif at the 5′ end. The resulting PCR product then can be cloned into a pENTR-SD/D-TOPO vector (Invitrogen) and subsequently used in an LR recombination reaction (Gateway Technology, Invitrogen) with the pAM4418 vector. To test the pAM4418 vector, the *yemGFP* gene was amplified by PCR from pJS151 using the primer pair yemGFP_F/yemGFP_R and cloned as described above to make the plasmid pAM4418-yemGFP.

GenElute HP Plasmid Miniprep Kits (Sigma-Aldrich) were used for isolation of plasmid DNA from *E. coli* strains. Plasmids were digested with restriction endonucleases from New England BioLabs or other suppliers in buffers recommended by the suppliers. All plasmid constructs were first screened by restriction analyses, and one positive clone was confirmed by DNA sequencing. Sequences were deposited in GenBank under the following accession numbers: JN376076-JN376080.

### IPTG induction of the *trc* promoter in pAM4418-yemGFP

*Leptolyngbya* BL0902 wild type and derivatives harboring the plasmid pAM4418-yemGFP or only pAM4418 were grown in BG-11 liquid medium, diluted to an OD_750_ of 0.15, and grown as 25 ml samples in 125 ml flasks on a shaker under standard conditions. After two days, the cultures were supplemented with IPTG to final concentrations of 0.1, 0.2, 0.5, 1, 2, 5, and 10 mM for pAM4418-yemGFP and 1 mM for the control strains. The emission intensities of yemGFP from samples of the cultures were measured with a Tecan Infinite(R) M200 plate reader (TECAN) after induction for 0, 1.5, 3, 6, 12, 21, 24, 27, and 48 h. The excitation wavelength was set at 488 nm, and the emission was measured at 518 nm.

## References

[1] Ducat DC, Way JC, Silver PA (2011). Engineering cyanobacteria to generate high-value products.. Trends Biotechnol.

[2] Heidorn T, Camsund D, Huang HH, Lindberg P, Oliveira P (2011). Synthetic biology in cyanobacteria engineering and analyzing novel functions.. Methods Enzymol.

[3] Wada H, Gombos Z, Sakamoto T, Murata N (1992). Genetic manipulation of the extent of desaturation of fatty acids in membrane lipids in the cyanobacterium *Synechocystis* PCC6803.. Plant Cell Physiol.

[4] Sato N, Wada H, Wada H, Murata N (2009). Lipid biosynthesis and its regulation in cyanobacteria.. Lipids in photosynthesis: essential and regulatory functions.

[5] Osanai T, Azuma M, Tanaka K (2007). Sugar catabolism regulated by light- and nitrogen-status in the cyanobacterium *Synechocystis* sp. PCC 6803.. Photochem Photobiol Sci.

[6] Mendez-Perez D, Begemann MB, Pfleger BF (2011). Modular synthase-encoding gene involved in alpha-olefin biosynthesis in *Synechococcus* sp. strain PCC 7002.. Appl Environ Microbiol.

[7] Schirmer A, Rude M, Li X, Popova E, Del Cardayre S (2010). Microbial biosynthesis of alkanes.. Science.

[8] Herrero A, Flores E (2008). Cyanobacteria: Molecular Biology, Genomics and Evolution..

[9] Pearl HW, Whitton BA, Potts M (2000). Marine Plankton.. The ecology of cyanobacteria: their diversity in time and space.

[10] Flores E, Herrero A (2010). Compartmentalized function through cell differentiation in filamentous cyanobacteria.. Nat Rev Micro.

[11] Kumar K, Mella-Herrera RA, Golden JW (2010). Cyanobacterial heterocysts.. Cold Spring Harb Perspect Biol 2009.

[12] Kehoe DM (2010). Chromatic adaptation and the evolution of light color sensing in cyanobacteria.. Proc Natl Acad Sci U S A.

[13] Gao Q, Garcia-Pichel F (2011). Microbial ultraviolet sunscreens.. Nat Rev Microbiol.

[14] Mur LR, Skulberg OM, Utkilen H, Chorus I, Bartram J (1999). Cyanobacteria in the environment.. Toxic cyanobacteria in water: A guide to their public health consequences, monitoring and management.

[15] Elhai J (1994). Genetic techniques appropriate for the biotechnological exploitation of cyanobacteria.. J Appl Phycol.

[16] Flores E, Muro-Pastor AM, Meeks JC, Herrero A, Flores E (2008). Gene transfer to cyanobacteria in the laboratory and in nature.. The cyanobacteria: molecular biology, genomics and evolution.

[17] Clerico EM, Ditty JL, Golden SS (2007). Specialized techniques for site-directed mutagenesis in cyanobacteria.. Methods Mol Biol.

[18] Koksharova OA, Wolk CP (2002). Genetic tools for cyanobacteria.. Appl Microbiol Biotechnol.

[19] Deng MD, Coleman JR (1999). Ethanol synthesis by genetic engineering in cyanobacteria.. Appl Environ Microbiol.

[20] Atsumi S, Higashide W, Liao JC (2009). Direct photosynthetic recycling of carbon dioxide to isobutyraldehyde.. Nature Biotechnol.

[21] Roessler PG, Chen Y, Liu B, Dodge CN (2009). Secretion of fatty acids by photosynthetic microorganisms.. US patent.

[22] Toyomizu M, Suzuki K, Kawata Y, Kojima H, Akiba Y (2001). Effective transformation of the cyanobacterium *Spirulina platensis* using electroporation.. J Appl Phycol.

[23] Kawata Y, Yano S, Kojima H, Toyomizu M (2004). Transformation of *Spirulina platensis* strain C1 (*Arthrospira* sp. PCC9438) with Tn*5* transposase-transposon DNA-cation liposome complex.. Mar Biotechnol.

[24] Rippka R, Deruelles J, Waterbury JB, Herdman M, Stanier RY (1979). Generic assignments, strain histories and properties of pure cultures of cyanobacteria.. J Gen Microbiol.

[25] Elhai J, Vepritskiy A, Muro-Pastor AM, Flores E, Wolk CP (1997). Reduction of conjugal transfer efficiency by three restriction activities of *Anabaena* sp. strain PCC 7120.. J Bacteriol.

[26] Castenholz RW, Rippka R, Herdman M, Wilmotte A, Boone DR, Castenholz RW, Garrity GM (2001). Form-genus V. *Leptolyngbya* Anagnostidis and Komárek 1988.. Bergey' Manual of Systematic Bacteriology.

[27] Anagnostidis K, Komárek J (1988). Modern approach to the classification system of cyanophytes. 3 - Oscillatoriales.. Arch Hydrobiol, Suppl 80.

[28] Sheng J, Vannela R, Rittmann BE (2011). Evaluation of methods to extract and quantify lipids from *Synechocystis* PCC 6803.. Bioresour Technol.

[29] Shestakov S, Khyen N (1970). Evidence for genetic transformation in blue-green alga *Anacystis nidulans*.. Mol Gen Genet.

[30] Wolk CP, Vonshak A, Kehoe P, Elhai J (1984). Construction of shuttle vectors capable of conjugative transfer from *Escherichia coli* to nitrogen-fixing filamentous cyanobacteria.. Proc Natl Acad Sci U S A.

[31] Tsinoremas NF, Kutach AK, Strayer CA, Golden SS (1994). Efficient gene transfer in *Synechococcus* sp. strains PCC 7942 and PCC 6301 by interspecies conjugation and chromosomal recombination.. J Bacteriol.

[32] Thomas CM, Smith CA (1987). Incompatibility group P plasmids: genetics, evolution, and use in genetic manipulation.. Annu Rev Microbiol.

[33] Billi D, Friedmann EI, Helm RF, Potts M (2001). Gene transfer to the desiccation-tolerant cyanobacterium *Chroococcidiopsis*.. J Bacteriol.

[34] Cohen MF, Wallis JG, Campbell EL, Meeks JC (1994). Transposon mutagenesis of *Nostoc* sp. strain ATCC 29133, a filamentous cyanobacterium with multiple cellular differentiation alternatives.. Microbiology.

[35] Ditta G, Stanfield S, Corbin D, Helinski DR (1980). Broad host range DNA cloning system for gram-negative bacteria: construction of a gene bank of *Rhizobium meliloti*.. Proc Natl Acad Sci U S A.

[36] Koksharova OA, Wolk CP (2002). A novel gene that bears a DnaJ motif influences cyanobacterial cell division.. J Bacteriol.

[37] Frazao B, Martins R, Vasconcelos V (2010). Are known cyanotoxins involved in the toxicity of picoplanktonic and filamentous North Atlantic marine cyanobacteria?. Mar Drugs.

[38] Komárek J, Anagnostidis K, Büdel B, Gärtner G, Krienitz L, Schagerl M (2005). Cyanoprokaryota 2.. Heidelberg: Elsevier GmbH, Spektrum Akademischer Verlag..

[39] Taton A, Grubisic S, Brambilla E, De Wit R, Wilmotte A (2003). Cyanobacterial diversity in natural and artificial microbial mats of Lake Fryxell (McMurdo Dry Valleys, Antarctica): a morphological and molecular approach.. Appl Environ Microbiol.

[40] Ewing B, Hillier L, Wendl MC, Green P (1998). Base-calling of automated sequencer traces using phred. I. Accuracy assessment.. Genome Res.

[41] Ewing B, Green P (1998). Base-calling of automated sequencer traces using phred. II. Error probabilities.. Genome Res.

[42] Gordon D (2003). Viewing and editing assembled sequences using Consed.. Curr Protoc Bioinform.

[43] Ludwig W, Strunk O, Westram R, Richter L, Meier H (2004). ARB: a software environment for sequence data.. Nucleic Acids Res.

[44] Pruesse E, Quast C, Knittel K, Fuchs BM, Ludwig W (2007). SILVA: a comprehensive online resource for quality checked and aligned ribosomal RNA sequence data compatible with ARB.. Nucleic Acids Res.

[45] Schloss PD, Westcott SL, Ryabin T, Hall JR, Hartmann M (2009). Introducing mothur: open-source, platform-independent, community-supported software for describing and comparing microbial communities.. Appl Environ Microbiol.

[46] Talavera G, Castresana J (2007). Improvement of phylogenies after removing divergent and ambiguously aligned blocks from protein sequence alignments.. Syst Biol.

[47] Guindon S, Gascuel O (2003). A Simple, fast, and accurate algorithm to estimate large phylogenies by maximum likelihood.. Syst Biol.

[48] Felsenstein J (2005). PHYLIP (Phylogeny Inference Package) version 3.6.. Distributed by the author Department of Genome Sciences, University of Washington, Seattle.

[49] Drummond AJ, Rambaut A (2007). BEAST: Bayesian evolutionary analysis by sampling trees.. BMC Evol Biol.

[50] Bligh EG, Dyer WJ (1959). A rapid method of total lipid extraction and purification.. Can J Biochem Physiol.

[51] Elhai J, Wolk CP (1988). Conjugal transfer of DNA to cyanobacteria.. Meth Enzymol.

[52] Liu D, Golden JW (2002). *hetL* overexpression stimulates heterocyst formation in *Anabaena* sp. strain PCC 7120.. J Bacteriol.

[53] Wu X, Lee DW, Mella RA, Golden JW (2007). The *Anabaena* sp. strain PCC 7120 *asr1734* gene encodes a negative regulator of heterocyst development.. Mol Microbiol.

[54] Huang G, Fan Q, Lechno-Yossef S, Wojciuch E, Wolk CP (2005). Clustered genes required for the synthesis of heterocyst envelope polysaccharide in *Anabaena* sp. strain PCC 7120.. J Bacteriol.

[55] Wilson KJ, Sessitsch A, Corbo JC, Giller KE, Akkermans AD (1995). beta-Glucuronidase (GUS) transposons for ecological and genetic studies of rhizobia and other gram-negative bacteria.. Microbiology.

[56] Li R, Golden SS (1993). Enhancer activity of light-responsive regulatory elements in the untranslated leader regions of cyanobacterial *psbA* genes.. Proc Natl Acad Sci U S A.

[57] Nakamura K, Inouye M (1979). DNA sequence of the gene for the outer membrane lipoprotein of *E. coli*: an extremely AT-rich promoter.. Cell.

[58] Bahassi E, O'Dea M, Allali N, Messens J, Gellert M (1999). Interactions of CcdB with DNA gyrase. Inactivation of GyrA, poisoning of the gyrase-DNA complex, and the antidote action of CcdA.. J Biol Chem.

[59] Orosz A, Boros I, Venetianer P (1991). Analysis of the complex transcription termination region of the *Escherichia coli rrnB* gene.. Eur J Biochem.

[60] Castiglioni B, Rizzi E, Frosini A, Sivonen K, Rajaniemi P (2004). Development of a universal microarray based on the ligation detection reaction and 16S rRNA gene polymorphism to target diversity of cyanobacteria.. Appl Environ Microbiol.

[61] Siegesmund MA, Johansen JR, Karsten U, Friedl T (2008). *Coleofasciculus* Gen. Nov (Cyanobacteria): Morphological and molecular criteria for revision of the genus *Microcoleus* Gomont.. J Phycol.

[62] Taton A, Grubisic S, Ertz D, Hodgson DA, Piccardi R (2006). Polyphasic study of Antarctic cyanobacterial strains.. J Phycol.

[63] Taton A, Grubisic S, Balthasart P, Hodgson DA, Laybourn-Parry J (2006). Biogeographical distribution and ecological ranges of benthic cyanobacteria in East Antarctic lakes.. FEMS Microbiol Ecol.

[64] Li Z, Brand J (2007). *Leptolyngbya nodulosa* sp. nov. (Oscillatoriaceae), a subtropical marine cyanobacterium that produces a unique multicellular structure.. Phycologia.

[65] Ishida T, Yokota A, Sugiyama J (1997). Phylogenetic relationships of filamentous cyanobacterial taxa inferred from 16S rRNA sequence divergence.. J Gen Appl Microbiol.

[66] Janse I, Kardinaal WE, Agterveld MK, Meima M, Visser PM (2005). Contrasting microcystin production and cyanobacterial population dynamics in two *Planktothrix*-dominated freshwater lakes.. Environ Microbiol.

[67] Marquardt J, Palinska KA (2007). Genotypic and phenotypic diversity of cyanobacteria assigned to the genus *Phormidium* (Oscillatoriales) from different habitats and geographical sites.. Arch Microbiol.

[68] Gorelova OA, Kosevich IA, Baulina OI, Fedorenko TA, Torshkhoeva AZ (2009). Associations between the white sea invertebrates and oxygen-evolving phototrophic microorganisms.. Moscow Univ Biol Sci Bull.

[69] Wood SA, Rueckert A, Cowan DA, Cary SC (2008). Sources of edaphic cyanobacterial diversity in the Dry Valleys of Eastern Antarctica.. ISME J.

[70] Mutsuda M, Michel K-P, Zhang X, Montgomery BL, Golden SS (2003). Biochemical properties of CikA, an unusual phytochrome-like histidine protein kinase that resets the circadian clock in *Synechococcus elongatus* PCC 7942.. J Biol Chem.

[71] Cormack BP, Valdivia RH, Falkow S (1996). FACS-optimized mutants of the green fluorescent protein (GFP).. Gene.

[72] Zacharias DA, Violin JD, Newton AC, Tsien RY (2002). Partitioning of lipid-modified monomeric GFPs into membrane microdomains of live cells.. Science.

[73] Yoon HS, Golden JW (1998). Heterocyst pattern formation controlled by a diffusible peptide.. Science.

